# Vascular–Metabolic Risk Factors and Psychological Stress in Patients with Chronic Tinnitus

**DOI:** 10.3390/nu14112256

**Published:** 2022-05-28

**Authors:** Benjamin Boecking, Sven Klasing, Michael Walter, Petra Brueggemann, Amarjargal Nyamaa, Matthias Rose, Birgit Mazurek

**Affiliations:** 1 Tinnitus Center, Charité, Universitätsmedizin Berlin, 10117 Berlin, Germany; benjamin.boecking@charite.de (B.B.); sven.klasing@charite.de (S.K.); petra.brueggemann@charite.de (P.B.); nyamaa.amarjargal@charite.de (A.N.); 2 Institute of Clinical Chemistry and Laboratory Medicine, Universitätsmedizin Rostock, 18057 Rostock, Germany; michael.walter@med.uni-rostock.de; 3 Medical Department, Division of Psychosomatic Medicine, Charité, Universitätsmedizin Berlin, 10117 Berlin, Germany; matthias.rose@charite.de

**Keywords:** chronic tinnitus, blood parameters, biomarkers, perceived stress, vascular–metabolic risk

## Abstract

Little is known about molecular correlates of chronic tinnitus. We examined interrelationships between vascular–metabolic risk factors, perceived stress, and other routine blood values in patients with chronic tinnitus. Two-hundred patients (51% female) were screened for 49 blood parameters pertaining to vascular–metabolic risk, immune function, and redox processes. They further completed perceived stress- and tinnitus-related distress questionnaires. Following descriptive analyses, gender-specific sets of age- and tinnitus-severity-adjusted regression models investigated associations between perceived stress and blood parameters. Patients reported mildly elevated levels of perceived stress. Elevated levels of total cholesterol (65% and 61% of female and male patients, respectively), non-HDL-c (43/50%), LDL-c (56/59%), and lipoprotein_a (28/14%) were accompanied by high rates of overweight (99/100%) and smoking (28/31%). A low-level inflammatory state was accompanied by reduced reactive oxygen species (ROS)-neutralizing capacity (reduced co-enzyme Q10 and SOD1 levels). Most vascular risk factors were not correlated with perceived stress, except for fibrinogen (*ß* = −0.34) as well as C-reactive protein (*ß* = −0.31, *p* < 0.05) in men, and MCV (*ß* = −0.26, *p* < 0.05) in women. Interrelations between blood parameters and stress levels need to be investigated within psychobehavioural frameworks across varying distress levels. Alongside psychological interventions, a low-level inflammatory state may be a route for pharmacological therapeutics.

## 1. Introduction

Chronic tinnitus—a symptom of interrelated biopsychological contributions—denotes the conscious awareness of a tonal or composite noise without identifiable corresponding external acoustic source. Prevalence estimates vary widely and range from 5 to 43% [1,2]. Whilst the majority of people habituate to the symptom [3], a proportion of patients experience psychological distress, which appears to facilitate symptom chronification [4]—possibly through interactions of pre-existing psychological vulnerability and cognitive–affective reaction patterns following symptom onset [5,6,7].

Biomarker research for chronic tinnitus is still in its infancy [8,9,10,11,12], yet a role of vascular risk factors and inflammatory processes has been suggested [13,14]. Other studies investigated cytokine changes [15], mean platelet volumes (MPV), and neutrophil-to-lymphocyte ratios (NLR) with inconclusive results. Some studies reported increased MPV [16,17] or NLRs [17,18] in tinnitus patients, whereas other studies did not find any such differences [19] or reported lower MPV levels in tinnitus patients compared to controls [20]. A few studies further reported an association between zinc status and tinnitus severity [21,22].

‘Stress’ has been defined as “the quality of experience […] which, through either overarousal or underarousal, results in psychological or physiological distress” [23]. Tinnitus-correlated psychological distress has been described using a variety of terms, including tinnitus handicap [24], tinnitus severity [25], tinnitus-related distress [26], tinnitus disability [27], tinnitus annoyance [28], tinnitus bother [29], or tinnitus distress [30]. Additionally, “perceived stress”, i.e., the degree to which people appraise situations as stressful [31] considerably overlaps with tinnitus-related distress [5,15,32,33].

Emotional distress and tinnitus symptomatology are intricately connected: For instance, perceived stress can trigger sudden hearing loss and facilitate tinnitus onset or chronification [34,35,36]—likely against a backdrop of psychological vulnerability [37]. Moreover, some low-quality evidence suggests an etiological role of perceived stress in the development of some chronic pain conditions [38]—which may share pathophysiological variance with chronic tinnitus presentations [32,39,40]. Concomitant to the tinnitus-symptom, psychological distress may prevent habituation to the tinnitus sound—and thereby facilitate symptom chronification and maintain affective arousal [41]. Lastly, the tinnitus sound itself can act as a stressor—therein closing a vicious cycle between perceived stress and tinnitus exacerbation [35,42].

Regarding biological underpinnings of perceived stress, Juster et al. [40] proposed 26 putative biomarkers that included immune, vascular-metabolic and oxidative parameters that were also denoted as possible transdiagnostic markers across various psychological conditions [43,44,45]. In this vain, biomarker candidates that were identified in patients with chronic tinnitus, and that relate to cardiovascular, inflammatory, or immune-related processes [12], may or may not overlap with biomarkers of perceived stress. 

In this work, we thus explored interrelationships between perceived stress, vascular-metabolic risk factors, and routine blood parameters in patients with chronic tinnitus. First, we describe the obtained blood parameters relative to their reference ranges in our patient sample. Second, we investigate associations between ‘outstanding’ biomarkers and patients’ perceived stress levels—considering potential gender differences [46,47].

## 2. Materials and Methods

### 2.1. Participants

The present study reports questionnaire and blood parameter data from *n* = 200 patients with chronic tinnitus (51% female; *M*_age_ = 54.68; *SD* = 8.44) who (a) self-referred to the Tinnitus Center at Charité Universitätsmedizin Berlin between April 2016 and August 2017; (b) suffered from chronic tinnitus (lasting for >3 months); (c) were 18 years of age or older; and (d) completed, amongst other measures, the German Tinnitus- and Perceived Stress Questionnaire. Exclusion criteria included an inability to consent due to severe psychiatric or physical limitations, as well as a participation in any other research study. Upon arrival at the Tinnitus Center, participants provided blood samples (obtained via 1 × 2 mL, 1 × 6 mL EDTA, 2 × 4.5 mL lithium heparin, 2 × 4.5 mL serum, and 1 × 2.7 mL citrate tubes), underwent audiological testing (the results of which are reported elsewhere), and completed the psychological questionnaires. Ethical approval was obtained from Charité Universitätsmedizin Berlin (No: EA1/115/15). All research was performed in accordance with the Declaration of Helsinki and informed consent was obtained from all participants.

### 2.2. Measures

#### 2.2.1. Blood Index Values

The obtained blood samples were screened for a number of indices. The following (I) cellular immune response markers were obtained: leukocytes, lymphocytes (total), lymphocytes (%), monocytes (total), monocytes (%), neutrophils (total), neutrophils (%), immature granulocytes (total), immature granulocytes (%), eosinophils (total), eosinophils (%), basophils (total), and basophils (%). (II) Inflammatory response markers included cytokines (TNF-α, IL-6) and acute-phase Proteins (CRP, fibrinogen, ferritin, thrombocytes, MPV). (III) Hematological markers included hemoglobin, hematocrit, erythrocytes, MCV, RDW_CV, MCH, and MCHC. Measured (IV) (co-)enzymes comprised superoxide_dismutase_1, superoxide_dismutase_2, lipid_peroxidase, and ubiquinone (Q10). (V) Vascular-metabolic risk markers included total cholesterol, triglycerides, HDL-c, non-HDL-c, LDL-c, and lipoprotein_a. (VI) Liver function markers included albumin, GOT, GPT, and gamma_GT. (VII) Kidney function markers included GFR and creatinine. (VIII) Purine metabolism was indexed by uric acid. Lastly, (IX) vitamins, minerals, and trace elements included calcium, magnesium, zinc, selenium, and vitamin D3.

#### 2.2.2. Perceived Stress

Perceived stress was measured using the Perceived Stress Questionnaire (PSQ; [48,49]). ‘Tension’ explores tense disquietude, exhaustion, and lack of relaxation. ‘Worries’ assesses anxious concern for the future, and feelings of desperation and frustration. ‘Joy’ assesses positive feelings of challenge, joy, energy, and security, and ‘Demands’ assesses perceived environmental demands, such as lack of time, pressure, and overload. The scale consists of 20 items that are rated on a 4-point scale (1 = almost never; 2 = sometimes; 3 = often; 4 = almost always). All indices are linearly transformed to range from 0 to 100 and summed up to a total score for which joy is recoded.

#### 2.2.3. Tinnitus-Related Distress

The German version of the tinnitus questionnaire [50,51] assesses tinnitus-related psychological distress. It consists of 52 statements that are answered on a 3-point scale (0 = not true; 1 = partly true; 2 = true) across five subscales (cognitive and emotional burden, persistence of sound, hearing difficulties, sleep difficulties, and somatic complaints). Based on clinical and research considerations, we include only the total score in our analysis [52,53]. The total score includes 40 items, with two items being included twice, thus yielding a score from zero to 84. Biesinger et al. [54] suggested a cut-off of 46 points to distinguish high vs. low symptom burden, i.e., “decompensated” vs. “compensated” tinnitus. The scale’s test–retest reliability is good (total score: *r* = 0.94; [55]).

### 2.3. Statistical Analyses

Statistical analyses included descriptive and univariate regression analyses. All analyses were computed using IBM SPSS Statistics (v. 24). The significance level was set to α = 0.05.

### 2.4. Data Preparation

Following visual inspection of the data, “extreme outliers” (defined as featuring *z*-factor values of >3.29) were excluded for each blood parameter.

### 2.5. Descriptive Analyses

For the blood parameter data, patient values were categorized as ‘normal’, ‘increased’, or ‘decreased’ using gender-specific norm-reference values that were provided by two processing laboratories (Labor Berlin—Charité Vivantes GmbH, biovis Diagnostik MVZ GmbH). Next, frequency counts of women and men with ‘increased’ or ‘decreased’ values were computed for each blood parameter. 

### 2.6. Univariate Regression Analyses

To examine the impact of perceived- but not tinnitus-related distress on the measured blood parameters, we investigated gender-separate univariate regression models, with PSQ scores as independent variables, blood parameters as dependent variables, and age as well as tinnitus-related distress as covariates. Due to the scarcity of blood-parameter research, expected small effect sizes, and the conservativeness of the Bonferroni correction [56,57], we conducted separate regression analyses—thereby tolerating increased type-I error rates. Thus, our findings necessitate replication. 

## 3. Results

Table 1 features an overview of the sociodemographic information.

### 3.1. Descriptive Indices

Table 2 features descriptive results for the obtained psychological indices; Table 3 for vascular-metabolic risk factors, and Table 4 for vascular-metabolic blood parameters.

No gender differences emerged. Lifetime histories and last-year incidents of vascular events were negligible (lifetime: coronary heart disease (*n* = 5); stroke (*n* = 3); cardiac insufficiency (*n* = 8); last-year: coronary heart disease (*n* = 2); stroke (*n* = 2); cardiac insufficiency (*n* = 5)).

Table 5 features means, standard deviations and reference values for non-vascular-metabolic blood parameters.

High proportions of both female and male patients showed decreased levels of superoxide–dismutase 1, lipid-corrected ubiquinone (Q10), and GFR, as well as increased levels of total cholesterol, non-HDL-c, and LDL-c. High proportions of female patients showed increased levels of lipoprotein_a, whilst high proportions of male patients showed increased levels of monocytes and decreased levels of zinc.

### 3.2. Associations between Perceived Stress and Blood Parameters

Age- and tinnitus-related distress-adjusted univariate regression analyses revealed associations between perceived stress and oxidative stress markers predominantly in female—and inflammatory and immunological markers predominantly in male patients with chronic tinnitus.

Specifically, the observed findings included MCV (positive association (+) with perceived stress, i.e., PSQ-total) in women, and CRP (negative association (−) with PSQ-total), fibrinogen (−), selenium (+), GPT (−), and basophils (+) in men.

Investigating subdimensions of perceived stress, the red cell number and volume-increasing stress response values with a reduction in MCHC were confirmed for both women and men. PSQ-worries predicted ferritin (+), MCV (+), MCHC (−), hematocrit (+), magnesium (+), zinc (+), superoxide–dismutase 2 (+), and lipoprotein_a (+) in women; and fibrinogen (−), MCV (+), MCHC (−), basophils (+), leukocytes (−), and neutrophils (−) in men. PSQ-tension predicted ferritin (+), MCV (+), hematocrit (+), hemoglobin (+), RDW-CV (−), zinc (+), and superoxide–dismutase 2 (+) in women; and fibrinogen (−), uric acid (−), MCHC (−), lipid-corrected ubiquinone (Q10) (−), and basophils (+) in men. PSQ-joy predicted ferritin (+), MCV (+), hematocrit (+), hemoglobin (+), RDW-CV (−), and zinc (+) in women; and MCV (+), MCHC (−), and IL-6 (+) in men. Finally, PSQ-demands predicted ferritin (+), MCV (+), MCHC (−), hematocrit (+), hemoglobin (+), superoxide–dismutase 2 (+), selenium (+), magnesium (+), and zinc (+) in women; and ferritin (−), uric acid (−), lipid-corrected ubiquinone (Q10) (−), and basophils (+) in men.

See Table 6 for an overview. Supplementary Figure S1 provides a visual conspectus.

## 4. Discussion

The present study sought to investigate (1) vascular-metabolic risk factors and blood parameters in patients with chronic tinnitus and (b) their associations with perceived stress.

### 4.1. Vascular–Metabolic Risk Factors

Substantial proportions of patients showed elevated levels of metabolic–vascular risk factors, including total cholesterol (women/men) (64.7/61.2%), non-HDL-c (43.1/50.0%), and LDL-c (55.9/59.2%), frequently within a context of overweight (BMI ≥ 26 for 44.6%/47.6%). Plasma levels of lipoprotein (a) were increased in female patients only (27.5%). Out of keeping with previous findings in patients with chronic tinnitus, IL-6, IL-10, and TNF-α-levels were not elevated in our sample—nor did patients yield elevated rates of mean platelet volumes or neutrophil-to-lymphocyte ratios.

Considering both direct medical and indirect psychobehavioural pathways, these metabolic markers may reflect either (a) primary pathophysiological factors contributing to chronic tinnitus (i.e., through vascular or inflammatory processes that may affect otological processes [58,59,60,61]) or (b) secondary factors that might be associated with patients’ attempts to regulate distressing psychological states, such as ‘unhealthy’ dietary intake or sedentary behaviors [62,63,64,65,66].

Overall, our findings point to an unfavorable vascular-metabolic situation in these chronic tinnitus patients that may require special monitoring. Future studies will have to investigate whether pharmacological treatment of vascular risk, inflammation [67] or enzymatic dysregulation [68,69] might be helpful for some patients. Any such approaches, however, ought to consider wider emotional and psychobehavioural influences [70].

### 4.2. Oxidative Stress

Previous studies suggested possible interrelationships of oxidative stress- and mental-health-related processes, as well as a higher degree of oxidative stress and reduced antioxidative capacity in patients with chronic tinnitus [21]. Moreover, in patients with chronic tinnitus and hearing loss, oxidative stress has been suggested to potentially contribute to tinnitus onset through facilitating hair cell death or cochlear damage [71,72].

In the present study, redox parameters were frequently depleted in both female and male tinnitus patients (superoxide–dismutase 1 [98.0/91.8%] and ubiquinone [Q10] [43.1/33.7%]). Plasma levels of zinc were decreased in men only (30.6%). As a possible explanation for a loss of antioxidant factors, patients’ kidney function (GFR) was decreased in 50% of women and 51% of men. A higher relative proportion of monocytes was observed in 24.5% of men and in 10.8% of the women.

Superoxide-dismutase is a key antioxidant enzyme, which defends cells against oxidative stress. Ubiquinone (Q10) functions as an antioxidant co-enzyme by preventing lipid peroxidation in mitochondria or cell membranes [73,74]. Heightened oxidative stress and impaired defense processes might facilitate inflammatory responses or mitochondrial dysfunction, ultimately influencing neurotransmission and clinical symptom presentations [75,76].

Oxidative stress has been associated with a variety of psychological conditions, including depression [77,78,79], post-traumatic difficulties [80], and psychosis-spectrum conditions [81]. For the latter, the ‘oxidative stress hypothesis’ suggests that oxidative damage to lipids, proteins, or DNA might be associated with self-perpetuating changes in enzymatic and non-enzymatic antioxidant systems that may mediate “behavioral and molecular anomalies … associated with schizophrenia” [82]. Some evidence further suggests that superoxide–dismutase may be a trait- rather than a state marker for psychotic-spectrum experiences, as both acutely relapsed and stable outpatients were found to yield decreased levels of this enzyme [75]. Lower iron levels (e.g., zinc)—as found in men in the present sample—have further been shown to facilitate oxidative stress and potentially associated inflammatory processes [83], mood-related difficulties [84], or noise-triggered stress responses [85]. Seen from this perspective, previous studies that reported an association between zinc and tinnitus severity [21,22] may be interpreted within an oxidative stress framework that associates oxidative stress markers with audiologically triggered or general psychological distress in the experiences of chronic tinnitus or psychosis-spectrum conditions. Whilst previous research has attempted to delineate tinnitus from acoustic hallucinations by contrasting the former as ‘unorganized’ [86,87,88], other researchers have argued for a joint conceptualization [89].

Overall, the observed reductions in oxidative stress markers may reflect psychobiological states that underlie transdiagnostically relevant subjective experiences of perceived stress. Speculatively, oxidative stress might confer a trait vulnerability to psychoaudiological misperception or mood-mediated inflammatory epiphenomena of psychological distress in patients with chronic tinnitus or other psychological conditions.

### 4.3. Perceived Stress

Age- and tinnitus-related distress-adjusted linear regression analyses investigated the extent to which perceived stress influenced blood parameters in female and male patients with chronic tinnitus. As shown in Table 6 (Figure S1), we observed only marginal associations with the blood parameter conspicuities described before. Rather, associations with perceived stress were found for blood parameters within their respective reference ranges. Whilst somewhat unexpected, this finding is to be interpreted in the context of only mild overall elevation of perceived stress in the current sample.

Overall, women but not men showed positive associations between perceived stress and oxidative stress and anemic markers, whilst men but not women showed signs of worry-associated immunosuppressive processes, as reflected in inverse influences of perceived stress on fibrinogen and ferritin levels alongside positive associations with basophils [90]. Whilst preliminary, these data are in agreement with previous observations, according to which perceived stress may influence hematocrit, mean corpuscular volume (MCV), mean corpuscular hemoglobin concentration (MCHC), and red blood cell distribution width (RDW_CV; [91]) values. Moreover, these results are in keeping with previous research suggesting that chronic stress exposure might be associated with oxidative damage in women [92], particularly if associated with maladaptive lifestyle behaviors [93]. Positive associations between perceived stress and hematological markers were previously observed in healthy individuals [91,94,95], and have also been linked to acoustic trauma [96]—suggesting possibilities of direct or indirect (i.e., stress-mediated) effects of aversive noise exposure on hematological markers. Contrary to expectations, we observed negative associations between perceived stress levels and inflammatory markers in men but not women. Whilst the majority of published studies report positive associations between perceived stress and inflammatory markers [97,98], some small studies reported inverse relations in the context of heightened vascular risk [99]. The reasons for these gender differences remain mostly unknown [100], and future studies are needed to replicate and extend on these findings.

The present study has important limitations. First, the blood parameters were only measured once, and the study design disallowed for the investigation of time-related fluctuations, comparisons with healthy or non-tinnitus control groups, or effects of psychological or pharmacological interventions. Second, both the investigated index symptom (‘chronic tinnitus’) and the observed metabolic effects may be caused or confounded by a large number of unmeasured influences. Third, whilst we computed separate linear regression models, biomarkers are likely woven into complex interaction networks that need to be identified and investigated jointly in high-powered studies [101]. Therefore, the presented data must be interpreted as exploratory—offering preliminary pointers for possible candidate biomarkers of perceived stress in chronic tinnitus. Moreover, future studies ought to more carefully control sample characteristics, thereby allowing for in-detail differentiations of the effects of perceived stress on biomarkers across various levels of emotional distress and related constructs such as anxiety or depression. Notwithstanding, the current study features a relatively large clinical sample, and provides first indications of vascular–metabolic risk alongside immunosuppressive effects in patients with chronic tinnitus.

## 5. Conclusions

The results of the present study highlight possible interactions between vascular-metabolic risk factors and perceived stress, which may have a reinforcing role in facilitating or maintaining chronic tinnitus symptomatology. Respective pathways, however, are unclear. To prevent or treat perceived stress-related phenomena, including, but not limited to chronic tinnitus, treatment planning ought to apply a multidisciplinary view with psychological and medical professionals intervening at the intersection of vascular–metabolic risk factors, oxidative-stress-related influences, and psychological affective-behavioural (lifestyle-related) influences, whose functions need to be individually conceptualized [44,102,103,104].

## Figures and Tables

**Table 1:** Sociodemographic data and patient characteristics (*n* = 169).

	*n*	%
Nationality		
German	158	79.0
Other	11	5.5
Education		
Completed junior apprenticeship	44	22.0
Completed senior apprenticeship	28	14.0
University degree	90	45.0
Employment ‘yes’	119	59.5
Relationship status		
Single	31	15.5
Married	114	57.0
Divorced	16	8.0
Widowed	7	3.5
Duration of tinnitus		
<0.5 year	21	10.5
0.5–1 year	25	12.5
1–2 years	12	6.0
2–5 years	31	15.5
>5 years	66	33.0
Past psychotherapy ‘yes’	86	43.0

**Table 2:** Means and standard deviations for the psychological indices.

	Total (*n* = 200)		Women (*n* = 102)		Men (*n* = 98)	
	Mean	*SD*	Mean	*SD*	Mean	*SD*
PSQ total	50.27	13.19	51.44	12.16	49.07	14.16
Worries	13.62	9.32	13.79	10.04	13.44	8.56
Tension	19.14	16.50	19.99	18.03	18.26	14.79
Joy *	16.80	15.67	15.89	13.31	17.74	17.83
Demands	17.08	14.55	17.46	15.33	16.70	13.76
TQ total	43.80	19.01	45.29	18.27	42.26	19.72

* Reversely coded (i.e., the higher the better). PSQ = Perceived Stress Questionnaire; TQ = Tinnitus Questionnaire (German version).

**Table 3:** Means and standard deviations of vascular–metabolic risk factors and frequency rates of blood parameters with increased or decreased incidence rates of ≥25%. Relevant indices are highlighted for emphasis.

		Total		Women		Men	
	Unit	Mean	*SD*	Mean	*SD*	Mean	*SD*
§ Weight	kg	78.86	15.71	78.59	16.04	79.12	15.50
§ BMI	kg/m^2^	26.32	4.44	25.94	4.70	26.70	4.17
		Frequency	% (total)	Frequency	% (women)	Frequency	% (men)
† Current smoking	‘yes‘	59	45.4	33	50.8	26	40.0
†§ Regular drinking	‘yes‘	29	22.3	14	21.5	15	23.1

§ These risk factors were only available for a subset of *n* = 128 patients (65% of total sample; *n* = 65 women; *n* = 63 men).† Operationalized as drinking regularly “at least weekly”. BMI = Body Mass Index (underweight: <18.5; normal: 18.5–25; overweight: 25–30; obese: >30).

**Table 4:** Frequency rates of participants with increased or decreased vascular-metabolic blood parameters. Indices with ‘outstanding’ rates of ≥ 25% are highlighted for emphasis.

	Unit	Mean	*SD*	Reference Values		Frequency Decreased						Frequency Increased					
				Men	Women	Total	(% Total)	Women	(% Women)	Men	(% Men)	Total	(% Total)	Women	(% Women)	Men	(% Men)
**Total cholesterol**	mg/dL	212.31	38.24	<200	<200	-	-	-	-	-	-	126	**(63.0)**	66	**(64.7)**	60	**(61.2)**
Triglycerides	mg/dL	124.25	62.24	≤200	≤200	-	-	-	-	-	-	23	(11.5)	7	(6.9)	16	(16.3)
HDL-c	mg/dL	63.23	18.34	≥35	≥45	6	(3)	3	(2.9)	3	(3.1)	-	-	-	-	-	-
**Non-HDL-c**	mg/dL	149.34	39.75	<150	<150	-	-	-	-	-	-	93	**(46.5)**	44	**(43.1)**	49	**(50.0)**
**LDL-c**	mg/dL	137.10	34.56	<130	<130	-	-	-	-	-	-	115	**(57.5)**	57	**(55.9)**	58	**(59.2)**
**Lipoprotein_a**	nmol/L	45.83	65.34	<72.0	<72.0	-	-	-	-	-	-	42	(21.0)	28	**(27.5)**	14	(14.3)

HDL-c = high-density lipoprotein; LDL-c = low-density lipoprotein; non-HDL-c = non-high-density lipoprotein.

**Table 5:** Means, standard deviations, reference values, and frequency rates of participants with increased or decreased non-vascular-metabolic blood parameters. Indices with ‘outstanding’ rates of ≥25% are highlighted for emphasis.

	Unit	Mean	*SD*	Reference Values		Frequency Decreased						Frequency Increased					
				Men	Women	Total	% Total	Women	% Women	Men	% Men	Total	% Total	Women	% Women	Men	% Men
Leukocytes	nL	6.58	1.58	3.9–10.5	3.9–10.5	6	(3)	3	(2.9)	3	(3.1)	2	(1.0)	-	-	2	(2.0)
Lymphocytes	absolute/nL	1.96	0.56	1.10–4.50	1.10–4.50	8	(4)	5	(4.9)	3	(3.1)	2	(1.0)	-	-	-	-
Lymphocytes	%	30.43	7.76	20.0–44.0	20.0–44.0	17	(8.5)	9	(8.8)	8	(8.2)	12	(6.0)	9	(8.8)	3	(3.1)
Monocytes	absolute/nL	0.52	0.16	0.10–0.90	0.10–0.90	-	-	-	-	-	-	6	(3.0)	1	(1.0)	5	(5.1)
**Monocytes**	%	7.94	1.92	2.0–9.5	2.0–9.5	-	-	-	-	-	-	35	(17.5)	11	(10.8)	24	**(24.5)**
Neutrophils	absolute/nL	3.87	1.20	1.50–7.70	1.50–7.70	1	(0.5)	1	(1.0)	-	-	-	-	-	-	-	-
Neutrophils	%	58.35	8.74	42.0–77.0	42.0–77.0	9	(4.5)	6	(5.9)	3	(3.1)	5	(2.5)	4	(3.9)	1	(1.0)
NLR	cells/μL	2.10	0.86	1–3 *	1–3 *	-	-	-	-	-	-	-	-	-	-	-	-
Immature_granulocytes	absolute/nL	0.02	0.02	<0.050	<0.050	-	-	-	-	-	-	11	(5.5)	1	(1.0)	10	(10.2)
Immature_granulocytes	%	0.36	0.20	0.0–1.0	0.0–1.0	-	-	-	-	-	-	4	(2.0)	-	-	4	(4.1)
Eosinophils	absolute/nL	0.14	0.10	0.02–0.50	0.02–0.50	3	(1.5)	1	(1.0)	2	(2.0)	1	(0.5)	-	-	1	(1.0)
Eosinophils	%	2.07	1.32	0.5–5.5	0.5–5.5	11	(5.5)	8	(7.8)	3	(3.1)	6	(3.0)	3	(2.9)	3	(3.1)
Basophils	absolute/nL	0.05	0.02	0.00–0.20	0.00–0.20	-	-	-	-	-	-	-	-	-	-	-	-
Basophils	%	0.74	0.32	0.0–1.8	0.0–1.8	-	-	-	-	-	-	-	-	-	-	-	-
TNF-α	pg/mL	0.32	0.05	<8.1	<8.1	-	-	-	-	-	-	-	-	-	-	-	-
IL6	ng/L	1.83	1.12	≤7.0	≤7.0	-	-	-	-	-	-	3	(1.5)	1	(1.0)	2	(2.0)
CRP	mg/L	1.61	1.72	<5.0	<5.0	-	-	-	-	-	-	10	(5.0)	7	(6.9)	3	(3.1)
Fibrinogen	g/L	2.72	0.57	1.60–4.00	1.60–4.00	4	(2)	4	(3.9)	-	-	3	(1.5)	2	(2.0)	1	(1.0)
Ferritin	qg/L	128.29	93.09	30.0–400.0	13.0–150.0	4	(2.0)	2	(2.0)	2	(2.0)	19	(9.5)	14	(13.7)	5	(5.1)
Thrombocytes	nL	244.33	54.29	150–370	150–370	1	(0.5)	-	-	1	(1.0)	5	(2.5)	4	(3.9)	1	(1.0)
MPV	fl	10.68	1.00	7.0–12.0	7.0–12.0	-	-	-	-	-	-	19	(9.5)	7	(6.9)	12	(12.2)
Hemoglobin	g/dL	14.40	1.24	13.5–17.0	12.0–15.6	5	(2.5)	2	(2.0)	3	(3.1)	5	(2.5)	-	-	5	(5.1)
Hematocrit	l/L	0.43	0.04	0.395–0.505	0.355–0.455	6	(3.0)	3	(2.9)	3	(3.1)	6	(3.0)	3	(2.9)	3	(3.1)
Erythrocytes	pl	4.82	0.43	4.3–5.8	3.9–5.2	1	(0.5)	-	-	1	(1.0)	5	(2.5)	-	-	5	(5.1)
MCV	fl	88.33	3.60	80.0–99.0	80.0–99.0	2	(1.0)	1	(1.0)	1	(1.0)	-	-	-	-	-	-
RDW_CV	%	12.79	0.58	11.5–15.0	11.5–15.0	-	-	-	-	-	-	-	-	-	-	-	-
MCH	pg	29.89	1.27	27.0–33.5	27.0–33.5	3	(1.5)	2	(2.0)	1	(1.0)	-	-	-	-	-	-
MCHC	g/dL	33.83	0.94	31.5–36.0	31.5–36.0	5	(2.5)	4	(3.9)	1	(1.0)	3	(1.5)	1	(1.0)	2	(2.0)
**Superoxide–Dismutase 1**	ng/mL	63.89	4.53	77–531	77–531	190	**(95.0)**	100	**(98.0)**	90	**(91.8)**	-	-	-	-	-	-
Superoxide–Dismutase 2	ng/mL	58.93	15.52	>40	>40	16	(8.0)	16	(15.7)	-	-	-	-	-	-	-	-
Lipid_Peroxidase	μmol/L	64.10	79.66	<200	<200	-	-	-	-	-	-	11	(5.5)	10	(9.8)	1	(1.0)
**Q10 (lipid-corrected)**	μmol/mmol	0.23	0.07	>0.2	>0.2	77	**(38.5)**	44	**(43.1)**	33	**(33.7)**	-	-	-	-	-	-
Albumin	g/L	46.36	2.50	35.0–52.0	35.0–52.0	-	-	-	-	-	-	3	(1.5)	-	-	3	(3.1)
GOT	U/L	24.84	6.46	<50	<35	-	-	-	-	-	-	4	(2.0)	2	(2.0)	2	(2.0)
GPT	U/L	27.92	12.22	<41	<31	-	-	-	-	-	-	38	(19.0)	15	(14.7)	23	(23.5)
Gamma_GT	U/L	24.64	13.29	8–61	5–36	-	-	-	-	-	-	12	(6.0)	9	(8.8)	3	(3.1)
**GFR**	mL/min	84.43	8.46	>90	>90	101	**(50.5)**	51	**(50.0)**	50	**(51.0)**	-	-	-	-	-	-
Uric acid	mg/dL	4.93	1.23	3.6–8.2	2.3–6.1	1	(0.5)	-	-	1	(1.0)	5	(2.5)	3	(2.9)	2	(2.0)
Creatinine	mg/dL	0.85	0.15	0.70–1.20	0.50–0.90	1	(0.5)	1	(1.0)	-	-	1	(0.5)	1	(1.0)	-	-
Calcium	mmol/L	2.34	0.09	2.15–2.50	2.15–2.50	5	(2.5)	3	(2.9)	2	(2.0)	-	-	-	-	-	-
Magnesium	mmol/L	0.85	0.05	0.66–1.07	0.66–1.07	-	-	-	-	-	-	-	-	-	-	-	-
**Zinc**	qmol/L	12.63	1.77	12.0–26.0	9.0–22.0	31	(15.5)	1	(1.0)	30	**(30.6)**	-	-	-	-	-	-
Selenium	qmol/L	1.00	0.21	0.60–1.50	0.60–1.50	4	(2.0)	2	(2.0)	2	(2.0)	4	(2.0)	3	(2.9)	1	(1.0)
Vitamin D3	nmol/L	65.30	21.02	50.0–150.0	50.0–150.0	46	(23.0)	24	(23.5)	22	(22.4)	-	-	-	-	-	-

CRP = C-reactive_protein; GFR = glomerular filtration rate; GOT = glutamate–oxalacetate–transaminase; GPT = glutamate–pyruvate–transaminase; IL6 = interleukin-6; MCH = mean corpuscular/cellular hemoglobin; MCHC = mean corpuscular hemoglobin concentration; MCV = mean corpuscular/cell volume; MPV = mean platelet volume; NLR = neutrophil/lymphocyte ratio; RDW_CV = red blood cell distribution width; TNF-α = tumor necrosis factor alpha; * reference value obtained from www.mdcalc.com/neutrophil-lymphocyte-ratio-nlr-calculator#pearls-pitfalls (accessed on 2 April 2022).

**Table 6:** Age- and tinnitus-related distress-adjusted univariate regression analyses with perceived stress indices being regressed on blood parameter values for the total sample, female patients, and male patients with chronic tinnitus. Only significant associations are reported (* = *p* < 0.05; ** = *p* < 0.01; *** = *p* < 0.001).

**Total Sample**		**PSQ_Total**		**Worries**		**Tension**		**Joy**		**Demands**	
		***β***	***t* (3.194)**	***β***	***t* (3.194)**	***β***	***t* (3.194)**	***β***	***t* (3.194)**	***β***	***t* (3.194)**
Vascular risk markers	Lipoprotein_a			0.14	1.99 *						
Inflammatory markers	CRP	−0.19	−2.12 *			−0.15	−2.10 *				
	Fibrinogen					−0.16	−2.36 *				
	Ferritin	−0.19	−2.11 *								
	MCV			0.29	4.27 ***	0.25	3.58 ***	0.25	3.40 **	0.27	3.96 ***
	MCHC			−0.23	−3.27 **	−0.20	2.84 **	−0.20	−2.59 *	−0.21	−2.96 **
Oxidative stress markers	Ubiquinone(Q10)_lipid-corrected			−0.15	−2.08 *	−0.22	−3.14 **			−0.22	−3.09 **
	Selenium			0.17	2.29 *						
	Magnesium			0.15	2.10 *						
	Zinc			0.27	3.78 ***	0.25	3.52 **	0.22	2.85 **	0.24	3.40 **
Cellular immune reponse	Basophils (abs)									0.17	2.35 *
	Basophils (%)			0.16	2.25 *					0.18	2.52 *
**Female Patients**		**PSQ_total**		**Worries**		**Tension**		**Joy**		**Demands**	
		***β***	***t* (3.98)**	***β***	***t* (3.98)**	***β***	***t* (3.98)**	***β***	***t* (3.98)**	***β***	***t* (3.98)**
Vascular risk markers	Lipoprotein_a			0.21	1.98 *						
Inflammatory markers	Ferritin			0.25	2.50 *	0.27	2.86 **	0.21	2.18 *	0.22	2.28 *
	MCV	0.26	2.06 *	0.36	3.64 ***	0.29	3.01 **	0.21	2.05 *	0.34	3.57 **
	MCHC			−0.24	−2.30 *					−0.21	−2.12 *
	Hematocrit			0.22	2.19 *	0.28	2.91 **	0.29	2.95 **	0.30	3.20 **
	Hemoglobin					0.24	2.42 *	0.24	2.43 *	0.24	2.44 *
	RDW_CV					−0.21	−2.13 *	−0.20	−1.99 *		
Oxidative stress markers	Selenium									0.21	2.12 *
	Magnesium			0.22	2.23 *					0.20	2.07 *
	Zinc			0.34	3.35 **	0.32	3.23 **	0.22	2.17 *	0.33	3.39 **
	SOD-2			0.22	2.16 *	0.21	2.19 *			0.20	2.07 *
**Male Patients**		**PSQ_Total**		**Worries**		**Tension**		**Joy**		**Demands**	
		***β***	***t* (3.94)**	***β***	***t* (3.94)**	***β***	***t* (3.94)**	***β***	***t* (3.94)**	***β***	***t* (3.94)**
Inflammatory markers	CRP	−0.31	−2.35 *								
	Fibrinogen	−0.34	−2.63 *	−0.28	−2.84 **	−0.26	−2.60 *				
	Ferritin									−0.26	−2.54 *
	MCV			0.23	−2.32 *			0.29	2.69 **		
	MCHC			−0.24	2.34 *	−0.23	−2.32 *	−0.24	−2.16 *		
	IL-6							0.31	2.87 **		
	Uric acid					−0.24	−2.33 *			−0.28	2.74 **
Oxidative stress markers	Ubiquinone(Q10)_lipid-corrected					−0.24	−2.43 *			−0.24	−2.71 **
	Selenium	0.30	2.23 *								
Immunological markers	Basophils (abs)			0.22	2.13 *	0.20	1.98 *				
	Basophils (%)	0.38	2.91 **	0.35	3.53 **	0.27	2.77 **			0.27	2.68 **
	Leukocytes			−0.21	−2.03 *						
	Neutrophils (abs)			−0.24	−2.32 *						
	Neutrophils (%)			−0.22	−2.17 *						
Liver function	GPT	−0.28	−2.11 *								

CRP = C-reactive protein; MCV = mean corpuscular/cell volume; MCHC = mean corpuscular hemoglobin concentration; PSQ = Perceived Stress Questionnaire; RDW_CV = red blood cell distribution width; SOD-2 = Superoxide Dismutase 2; GPT = glutamate–pyruvate–transaminase; IL-6 = interleukin-6.

## Data Availability

As per Charité Universitaetsmedizin Berlin’s ethics committee, unfortunately we cannot make the data public without restrictions because we did not obtain patients’ consent to do so at the time. Nevertheless, interested researchers can contact the directorate of the Tinnitus Center Charité Universitaetsmedizin Berlin with data access requests (birgit.mazurek@charite.de) or the Charité’ s Open Data and Research Data Management Officer Dr. Evgeny Bobrov (evgeny.bobrov@charite.de).

## Supplementary Materials

The following supporting information can be downloaded at: https://www.mdpi.com/article/10.3390/nu14112256/s1: Figure S1: Perceived stress and blood parameters.
