# Supplementary figures and images for: Vascular–Metabolic Risk Factors and Psychological Stress in Patients with Chronic Tinnitus
Source: Nutrients. 2022 May 28;14(11):2256. doi: 10.3390/nu14112256 (PMC9183085; doi:10.3390/nu14112256)

**Figure S1.** Perceived Stress and Blood Parameters.

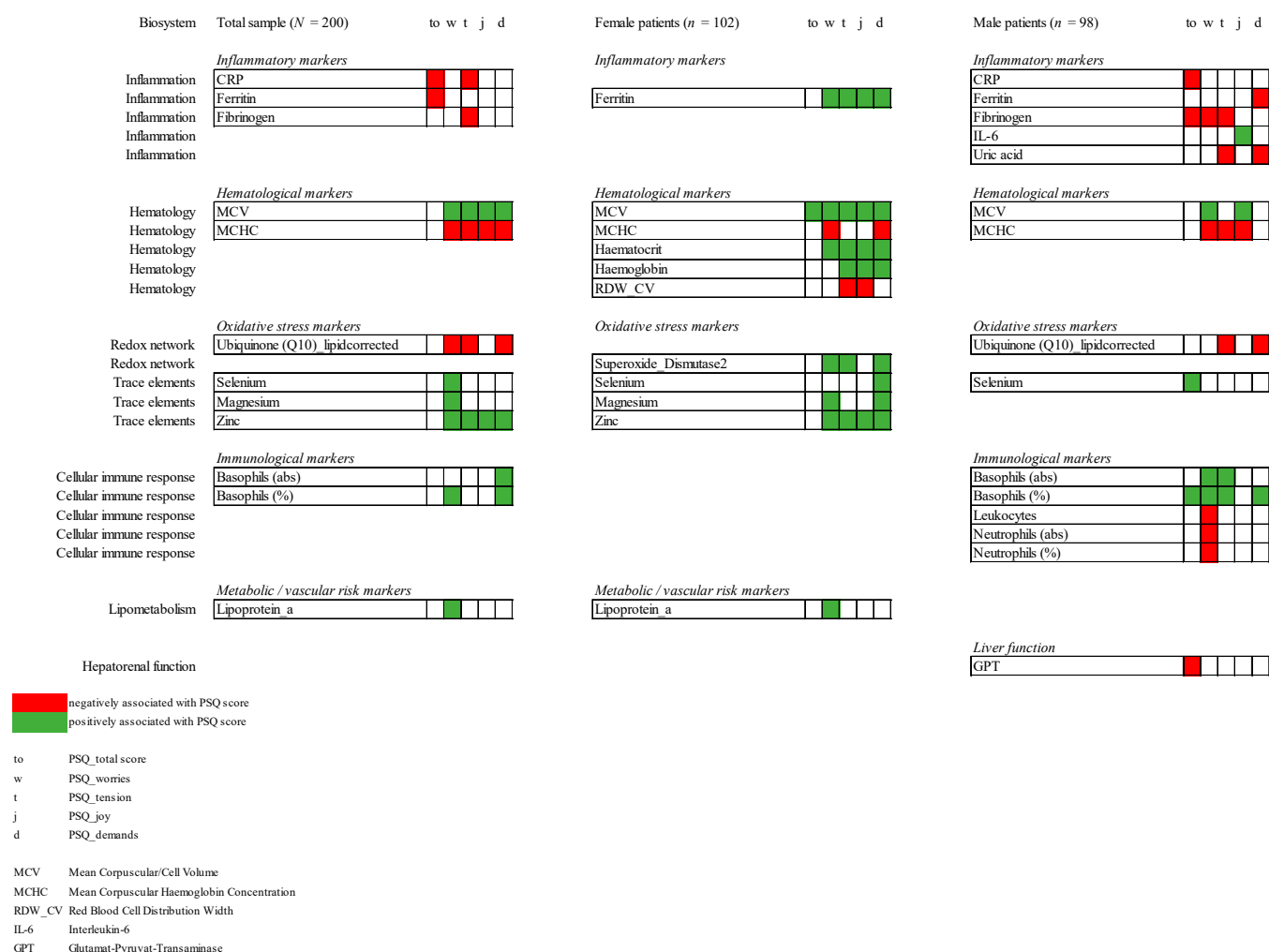

Supplement: Supplementary file 1 [file nutrients-14-02256-s001.zip › nutrients-1686983-supplementary.pdf]
